# Motor and cognitive changes in normal aging

**DOI:** 10.3389/fnagi.2014.00331

**Published:** 2014-11-25

**Authors:** Ahmed A. Moustafa

**Affiliations:** Department of Veterans Affairs, New Jersey Health Care System, School of Social Sciences and Psychology, Marcs Institute for Brain and Behaviour, University of Western SydneySydney, NSW, Australia

**Keywords:** aging, motor processes, cognitive processes, dopamine, acetylcholine, basal ganglia, hippocampus, prefrontal cortex

In a recent study, Cid-Fernandez et al. (2014) tested attentional performance in 3 age groups: young (21–29 years old), middle-aged (51–64 years old), and older adults (65–84 years old). The task used in this study involved presenting both visual and auditory cues to the participants, and they were required to pay attention to the visual cues while ignoring the auditory cues (for task details, see Escera et al., 1998). Cid-Fernandez et al. (2014) found that there was an increase in distractibility and changes in motor selection in the middle-aged and older groups, compared to the young group. These findings were revealed using electroencephalography analyses which related different cognitive and motor processes to different event-related potential components. Impairment in sensory filtering (e.g., longer time to characterize stimuli in working memory), as revealed by differences in the N2b component among the groups are presumed to explain the deficits in motor processes (i.e., selection of motor responses). It is thus suggested that these cognitive working memory changes in aging lead to slowing down of motor response selection.

Other prior studies have investigated changes in motor changes in aging (Light, 1990). In one study, it was reported that abnormalities in motor processing are not related to cognitive processing (Kolev et al., 2006). For example, Falkenstein et al. (2006) also reported abnormalities in motor selection processes in healthy aging. However, unlike the Cid-Fernandez et al. (2014) study did not find a different in early stimulus processing. Further, Falkenstein et al. (2006) have only tested younger and older adults. The differences in findings could be related to different age groups in both studies.

What is important about this study is Cid-Fernandez and colleagues (a) have tested both motor and cognitive processes in (b) three age groups. Most existing studies on aging often compare younger (aged 18–30 or 50 years old) with older (aged 60 or 65 years and over) adults (Scott, 1994; Braver and Barch, 2002; Cabeza et al., 2002; Fera et al., 2005; Weiler et al., 2008; Willemssen et al., 2011; Brehmer et al., 2012; Trewartha et al., 2014), and thus do not reveal the subtle motor and cognitive changes that may occur during the aging process, for example at 40s or 50s years old. Cid-Fernandez et al. (2014) found that attentional and motor changes can occur from 50 years onwards. Since Cid-Fernandez and colleagues did not recruit participants in their 40s, it is not known whether such cognitive and motor changes can occur at a younger age than reported by the authors. Similarly, studies that recruit only younger and older adults may not reveal the exact age (or age group) at which behavioral and neural changes occur.

Importantly, there are studies that test behavioral performance in more age groups than those recruited in the Cid-Fernandez et al. (2014) study (see for example studies by Davis et al., 2003, 2013; Krishna et al., 2012; Stark et al., 2013). One such study (Davis et al., 2003) recruited 4 groups: Group 1 (30–44 years old), Group 2 (45–59 years old), Group 3 (60–74 years old), Group 4 (75–90 years old). Using the Rey Auditory Verbal Learning Test (RAVLT, which measures memory acquisition, recall, and recognition), Davis et al. (2003) found that memory acquisition was lower in the 60–74 and 75–90 age groups, compared to the other groups. They also found that forgetting after one-day delay was more common in some, but not all, participants in the 75–90 years old group, in comparison to the other age groups. These findings suggest that impairment in cognitive performance occur at different times during the aging process, depending on the cognitive process being investigated as well as age group.

Other studies have recruited participants across various age groups and correlated behavioral performance with age (Davis et al., 2013; Stark et al., 2013). For example, Stark and colleagues reported impaired performance in hippocampus-based tasks in some aging populations (Stark et al., 2013; Bennett et al., 2014). One recent study by Davis and colleagues have recruited participants from age 5 to age 90 (Davis et al., 2013), which is probably among the most inclusive studies of age groups. Davis and colleagues found that verbal learning impairments were common among children and older adults in comparison to younger and middle-aged adults. The benefit of having such a wide range of age groups is to reveal at which age acquisition (in early ages) as well as decline (in older age) of motor and cognitive processes may occur.

Cid-Fernandez et al. (2014) suggest that their behavioral and physiological findings of impaired attentional performance can be related to changes in the prefrontal cortex during aging. This is supported by an extensive body of research showing impaired prefrontal function during the aging process (West, 1996; Braver and Barch, 2002; Solbakk et al., 2008; Wang et al., 2011; Johnson et al., 2013). In addition to the prefrontal cortex, healthy aging also affects the basal ganglia and hippocampus structures as well as neurotransmitters projecting to these brain regions, including dopamine and acetylcholine (Kaasinen et al., 2000; Inoue et al., 2001; Small et al., 2011). However, it is argued that brain changes (as well as corresponding behavioral decline) do possibly occur at different degrees and at different times during the aging process (Krishna et al., 2012). In one recent study, we found that older adults (75 and above) show more impairment in hippocampal-based tasks compared to less older adults (60–70 years old), but basal ganglia-based learning was impaired in the older and less older groups (Krishna et al., 2012; also see Moustafa et al., 2012). Thus, future work should explain how alterations to certain brain regions and neurotransmitters correspond to specific motor and cognitive changes in different age groups.

The Cid-Fernandez et al. (2014) findings have implications for the understanding of motor and cognitive problems associated with age-related neurodegenerative disorders, including Parkinson's and Alzheimer's diseases. Prior studies have also reported deficits in the selection of motor responses, yet it is not known whether these deficits are due to motor and/or cognitive abnormalities in these in different age-related neurodegenerative disorders (Hocherman et al., 2004; van Deursen et al., 2009).

## Conflict of interest statement

The author declares that the research was conducted in the absence of any commercial or financial relationships that could be construed as a potential conflict of interest.
